# A Case of AL Amyloidosis With Hepatomegaly as the Main Clinical Manifestation

**DOI:** 10.1002/ccr3.71640

**Published:** 2025-12-08

**Authors:** Shuchen Dong, Renjun Lv, Wei Wang, Guoling Gao, Mingfeng Zhao, Xingbin Ma

**Affiliations:** ^1^ Department of Gastroenterology and Hepatology Binzhou Medical University Hospital Binzhou Shandong Province China; ^2^ Department of Geriatrics Shandong Provincial Hospital Affiliated to Shandong First Medical University Jinan Shandong Province China; ^3^ Department of Pathology Binzhou Medical University Hospital Binzhou Shandong Province China

**Keywords:** AL amyloidosis, hepatomegaly, liver biopsy, prognosis

## Abstract

Light chain (AL) amyloidosis presenting predominantly with hepatomegaly is a rare and frequently misdiagnosed condition. While clinical features are non‐specific, elevated ALP/GGT should raise suspicion. A liver biopsy remains the gold standard for diagnosis. Although there is no disease‐specific therapy for hepatic amyloidosis, timely chemotherapy initiation—particularly monoclonal antibody‐based regimens—can significantly improve prognosis and lead to symptom resolution.

## Key Clinical Message

Light‐chain amyloidosis with primary hepatic involvement is rare and often misdiagnosed due to non‐specific clinical manifestations. This case illustrates that unexplained hepatomegaly with elevated ALP and GGT should necessitate a liver biopsy. Timely diagnosis and monoclonal antibody‐based therapy can markedly improve the patient's prognosis.

## 1 Introduction

Amyloidosis is a rare and heterogeneous group of diseases, most commonly associated with the accumulation of abnormal plasma cells. However, it also includes other forms such as transthyretin amyloidosis (both wild‐type and variant), serum amyloid A protein amyloidosis, leukocyte chemotactic factor‐2 amyloidosis, and several even rarer types. The hallmark of amyloidosis is the misfolding of immunoglobulin light or heavy chains, which form amyloid fibrils that are deposited in multiple organs or tissues throughout the body. The heart, kidneys, and peripheral nerves are most frequently affected [1]. Due to its complex and non‐specific clinical presentations, amyloidosis is frequently misdiagnosed, particularly in the early stages, leading to delays in treatment and adverse patient outcomes [2]. Although the liver is occasionally involved, it is exceedingly rare for the liver to be the primary organ affected [3]. Here, we report a patient who initially presented with fatigue, abdominal distension, and hepatomegaly, and was eventually diagnosed with light chain (AL) hepatic amyloidosis.

## 2 Case History/Examination

A 59‐year‐old male patient was admitted to the Department of Gastroenterology at the Affiliated Hospital of Binzhou Medical University in March 2023. The patient had no significant alcohol or hepatotoxic substance use and had no relevant family history. Eight months prior, he had experienced fatigue and abdominal distension without any other notable symptoms. An enhanced CT scan of the abdomen, conducted at a local hospital, revealed liver enlargement and poorly visualized hepatic veins. The initial diagnosis was “hepatic vein obstructive disease,” and the patient was referred to the interventional vascular surgery department of our hospital for further evaluation through angiography. However, angiography showed no significant abnormalities in the hepatic vein or inferior vena cava, effectively excluding hepatic veno‐occlusive disease. The patient was then admitted to our department.

## 3 Differential Diagnosis, Investigations and Treatment

To systematically rule out other potential causes of hepatomegaly, we conducted a series of investigations. By physical examination, the patient was of slim build (BMI 18.8 kg/m^2^), with no evidence of jaundice in the skin or sclera. The liver was palpably enlarged below the costal margin, presenting as firm, with regular borders and no tenderness, pulsation, or percussion pain. Laboratory tests showed the following results: total bilirubin (TBIL) 37.09 μmol/L (reference ranges: 5–23 μmol/L), direct bilirubin (DBIL) 17.02 μmol/L, ALP 325.10 U/L (35–135 U/L), γ‐GGT 569.80 U/L (7–45 U/L), AST 63.8 U/L (13–35 U/L), and ALT 37.8 U/L (7–40 U/L). Other parameters, including immunology, ceruloplasmin, and hepatotropic virology, were within normal limits. Echocardiography showed no abnormalities. An abdominal MRI revealed liver enlargement, particularly in the right lobe, and abnormal signals in both the liver and spleen. No significant abnormalities were observed in the bilateral kidneys. The findings effectively excluded infiltrative malignancies, including hepatocellular carcinoma, cholangiocarcinoma, and metastatic tumors. Systemic conditions such as sarcoidosis and lymphoproliferative disorders were also excluded. This stepwise exclusion of alternative etiologies provided a focused rationale for proceeding with liver biopsy.

**FIGURE 1:**
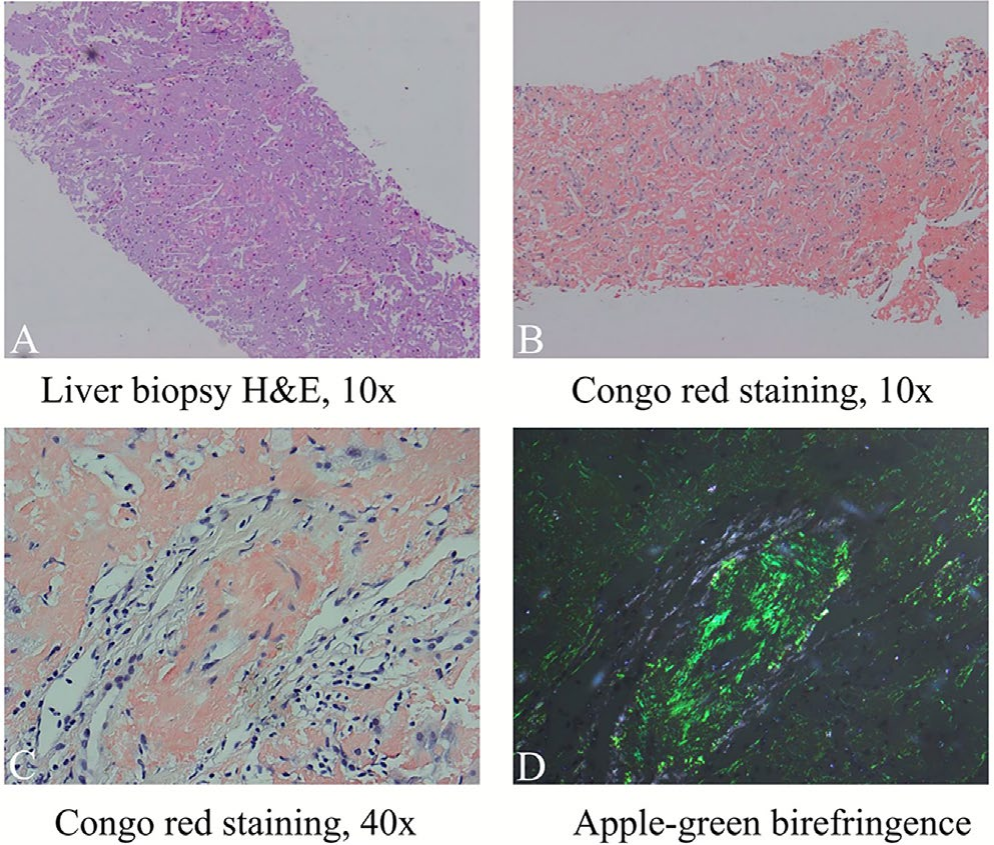
(A) Histologic section shows massive replacement of normal liver parenchyma by amorphous, eosinophilic, extracellular deposits (hematoxylin–eosin [H&E] staining; magnification × 10). (B, C) Congo red staining demonstrates amyloid deposition in the liver tissue. (D) Polarized light examination of a Congo red–stained section reveals classic apple‐green birefringence.

**FIGURE 2:**
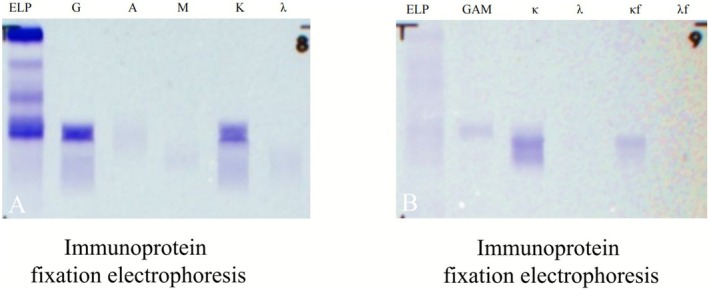
(A) Serum immunofixation electrophoresis demonstrates monoclonal IgG and kappa light chain. (B) Urine electrophoresis reveals Bence Jones protein corresponding to kappa free light chain.

Histopathological examination of the liver biopsy revealed homogeneous eosinophilic amyloid deposits in the perisinusoidal space. Congo red–positive deposits with apple‐green birefringence under polarized light, confirmed amyloidosis (Figure 1). To further subtype the deposits, immunohistochemical staining was performed, which demonstrated positivity for κ light chains and negativity for λ light chains. These findings were concordant with the systemic work‐up. Specifically, bone marrow biopsy and immunofixation electrophoresis revealed an IgG‐κ monoclonal band. Serum protein electrophoresis demonstrated an M‐protein level of 7.46 g/L. Serum free light chain assay showed elevated κ light chains at 163 mg/L (6.7–22.4 mg/L) and λ light chains at 7.62 mg/L (8.3–27.0 mg/L), with a κ/λ ratio of 21.39. Additionally, Bence Jones protein was detected in urine electrophoresis and identified as κ light chain type (Figure 2). Bone marrow aspiration revealed a 2.28% monoclonal plasma cell proportion, which did not meet the diagnostic criteria for multiple myeloma. Taken together, these results confirmed the diagnosis of AL hepatic amyloidosis with κ light chain predominance. The patient was initiated on monoclonal antibody–based chemotherapy in our hospital, including daratumumab (900 mg iv), bortezomib (1.3 mg/m^2^ sc), and dexamethasone (20 mg iv).

## 4 Conclusion and Results

**FIGURE 3:**
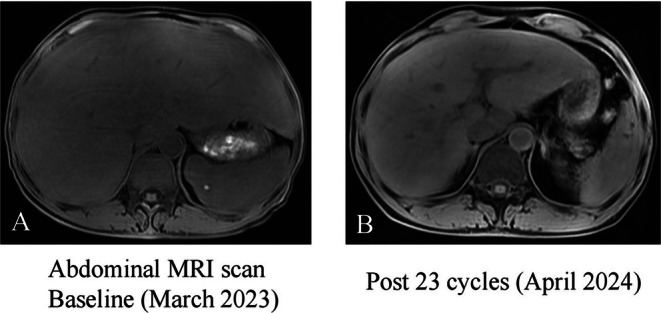
(A) Magnetic resonance (MR) imaging shows giant hepatomegaly prior to therapy initiation (baseline, March 2023). (B) Follow‐up MR imaging after 23 treatment cycles (April 2024) demonstrates marked reduction in liver size.

After 1 year of treatment, the patient showed significant symptomatic improvement. Follow‐up testing showed marked biochemical improvement, with liver enzymes and bilirubin levels nearly normalized (Table 1). MRI demonstrated a 10 cm reduction in liver transverse diameter (25 cm → 15 cm) and a decrease in liver volume from 4525.754 cm^3^ to 1884.499 cm^3^. The heterogeneous signal intensity also improved, with T1 values increasing from 1103.2 ms to 1522.2 ms, indicating significant radiologic recovery (Figure 3).

**TABLE 1:** Biochemical and radiologic changes before and after treatment in a patient with AL hepatic amyloidosis.

Parameter	Baseline (March 2023)	Follow‐up (September 2024)	Change
ALP (U/L)	325.10 ↑	113.20	↓ 211.9
γ‐GGT (U/L)	569.80 ↑	46.9 ↑	↓ 522.9
AST (U/L)	63.8 ↑	24.5	↓ 39.3
ALT (U/L)	37.8	27.7	↓ 10.1
TBIL (μmol/L)	37.09 ↑	28.76 ↑	↓ 8.33
DBIL (μmol/L)	17.02 ↑	4.41	↓ 12.61
Liver transverse diameter (cm)	25	15	↓ 10
Liver volume (cm^3^)	4525.754	1884.499	↓ 2641.255
Liver signal intensity (T1, ms)	1103.2	1522.2	↑ 419

Hepatic amyloidosis is typically characterized by hepatomegaly and elevated levels of ALP or GGT. Liver biopsy remains a cornerstone in establishing a definitive diagnosis. Isolated hepatic involvement correlates with significantly better survival than multiple organs. Although there is currently no disease‐specific therapy for amyloidosis, early diagnosis, timely intervention, and ongoing research are critical for improving patient outcomes and quality of life.

## 5 Discussion

Amyloidosis is a disorder characterized by the extracellular deposition of insoluble amyloid fibrils, which disrupt normal tissue architecture and function, ultimately leading to organ dysfunction [4]. Among the different forms of amyloidosis, primary amyloidosis, or AL amyloidosis, is the most prevalent. Alongside AL amyloidosis, heavy and light chain amyloidosis (AHL) and heavy chain amyloidosis (AH) are rarer subtypes. In AHL, amyloid fibrils are derived from both immunoglobulin heavy and light chain fragments, while in AH, amyloid deposits consist solely of heavy chain fragments [5].

Amyloidosis can present with a wide array of clinical symptoms, making diagnosis challenging. No single imaging, blood, or urine test can directly confirm the diagnosis, which increases the risk of misdiagnosis and delays in treatment. Approximately 70% of patients present with multiple organ involvement at the time of diagnosis, resulting in high mortality and poor long‐term prognosis [6]. The kidneys and heart are the organs most commonly affected. Renal involvement typically presents as nephrotic syndrome with progressive renal function dysfunction, and about 75% of patients develop proteinuria, with or without renal insufficiency [5]. When amyloid deposits occur in the heart, it can result in restrictive cardiomyopathy, often causing rapidly progressive heart failure [7]. However, it is crucial to note that amyloidosis can affect nearly any tissue in the body, excluding the brain [8].

The diverse clinical manifestations of amyloidosis, combined with the absence of a single confirmatory diagnostic test, highlight the importance of maintaining a high index of suspicion, particularly in patients with unexplained multi‐organ dysfunction. Increasing evidence emphasizes the evolving role of biomarkers in the early diagnosis and prognosis of systemic AL amyloidosis. In particular, a recent systematic review and meta‐analysis by Albulushi et al. demonstrated that cardiac biomarkers such as NT‐proBNP and troponins provide significant prognostic value in cardiac amyloidosis and can aid in the earlier detection of systemic disease [9]. Beyond cardiac markers, other tools such as serum amyloid P component, quantitative free light‐chain assays, and emerging multimodal imaging techniques have also shown promise in predicting disease progression and treatment response [10]. Together, these advances underscore the necessity of integrating biomarker evaluation with histopathology and imaging to optimize early diagnosis, risk stratification, and longitudinal monitoring of patients with AL amyloidosis. Liver involvement in amyloidosis is not uncommon; however, primary liver amyloidosis is rare, accounting for approximately 5% of cases [11]. When the liver is involved, clinical manifestations are typically mild due to the nonspecific nature of hepatic amyloid deposition. Symptoms most commonly include hepatomegaly, abdominal distension, fatigue, and anorexia. In some cases, portal hypertension, upper gastrointestinal bleeding and liver failure may occur.

Hepatomegaly in amyloidosis has both primary and secondary causes. The primary mechanism is the deposition of pathological fibrils derived from monoclonal immunoglobulin light and heavy chains in the liver tissue. These deposits are typically located in the peri‐sinusoidal space (space of Disse), the liver parenchyma, and within the vessel wall [12]. Secondary hepatomegaly is mainly attributable to right‐sided heart failure resulting from cardiac amyloid involvement, which leads to poor venous return congestion, and enlargement of the liver [13].

On physical examination, patients with hepatic amyloidosis often present with a firm, enlarged liver edge that is palpable but typically non‐tender. Laboratory findings are usually characterized by elevated ALP, often accompanied by increased γ‐GGT levels. In cases of liver failure or decompensated cirrhosis, additional abnormalities may be observed in coagulation parameters, bilirubin, and transaminases [14]. Imaging findings are generally nonspecific. The main manifestations on abdominal CT or MRI include hepatomegaly and diffuse low liver density, while contrast‐enhanced CT/MRI typically shows no significant abnormalities [15].

A definitive diagnosis of hepatic amyloidosis typically requires a liver biopsy. The pathological diagnosis is based on the β‐sheet structure of amyloid fibrils, which shows apple‐green birefringence under polarized light after Congo red staining [16]. In cases where a liver biopsy cannot be performed, PET‐CT using amyloid‐targeted tracers is emerging as a new tool for evaluating amyloidosis [17]. Additionally, bone marrow aspiration may be necessary to rule out multiple myeloma due to the low plasma cell burden in amyloidosis. Compared with this patient, the primary manifestations were liver enlargement, abdominal distension, and fatigue, accompanied by elevated ALP and GGT levels. Urine protein, creatinine and renal MR were normal, and ECG and cardiac ultrasound were also normal, which effectively ruled out kidney and heart involvement. Ultimately, AL‐type hepatic amyloidosis was diagnosed through liver and bone marrow biopsy, serum free light chain determination and related investigations.

With a growing understanding of amyloidosis and the advent of combination chemotherapy, patient survival and prognosis have improved significantly. In the case of AL amyloidosis, the primary treatment goal is to eliminate monoclonal light chain fragments produced by plasma cell clones. This reduction in serum light chain levels helps prevent further amyloid deposition in affected organs and tissues, ameliorates organ dysfunction, and provides more opportunities for recovery [18, 19]. A variety of treatment strategies are available for AL amyloidosis. High‐dose melphalan combined with autologous stem cell transplantation (HDM‐ASCT) has been considered for low‐risk patients. However, recent evidence indicates daratumumab‐bortezomib–based regimens as superior first‐line therapy [20]. This is backed by the ANDROMEDA trial (*N* = 361), which demonstrated that the Dara‐CyBorD regimen achieved a higher hematological very good partial response (VGPR) or better rate (60.8% vs. 31.1%; *p* < 0.001) compared to CyBorD [21]. In our patient's case, after 1 year of regular treatment, ALP levels decreased by more than 50% compared to baseline, and the liver's transverse diameter was reduced by 10 cm, demonstrating the safety and effectiveness of the daratumumab‐based regimen. Consistent with this, a recent study by Rees et al. analyzing 130 patients with hepatic AL amyloidosis reported that isolated hepatic involvement was associated with favorable hepatic responses and overall survival. The study highlighted that baseline ALP ≥ 4× ULN and achievement of hematologic response were independent predictors of hepatic response [22]. These findings align with the favorable outcome observed in our patient and underscore the importance of early diagnosis, close monitoring, and effective treatment in patients with isolated hepatic AL amyloidosis.

However, long‐term surveillance remains essential, as AL amyloidosis is a systemic and progressive disorder with the potential for multi‐organ involvement even after initial therapeutic response. Although our patient achieved marked biochemical and radiologic remission, sustained hematologic control is critical to improving survival and reducing the risk of relapse. Therefore, structured follow‐up should be emphasized. Regular monitoring of serum free light chains and liver function every 3–6 months is recommended to assess ongoing hematologic and hepatic response. In addition, annual cardiac MRI and echocardiography are warranted, given the high prevalence and prognostic impact of cardiac amyloidosis [23]. Renal function testing should also be performed periodically, as kidney involvement is common and often determines long‐term outcomes. Such a comprehensive surveillance strategy facilitates the early detection of organ progression, allows timely modification of therapy, and contributes to the optimization of long‐term management.

## Author Contributions

**Shuchen Dong:** writing – original draft. **Renjun Lv:** data curation. **Wei Wang:** resources. **Guoling Gao:** data curation. **Mingfeng Zhao:** formal analysis. **Xingbin Ma:** writing – review and editing.

## Funding

The authors have nothing to report.

## Ethics Statement

The studies involving humans were approved by the Ethics Committee of Binzhou Medical University Hospital. The studies were conducted in accordance with the local legislation and institutional requirements. Written informed consent was obtained from the individual(s) for the publication of any potentially identifiable images or data included in this article. Written informed consent was obtained from the participant/patient(s) for the publication of this case report.

## Consent

Written informed consent was obtained from the patient for the publication of this case report and any related images.

## Conflicts of Interest

The authors declare no conflicts of interest.

## Data Availability

The raw data supporting the conclusions of this article will be made available by the authors, without undue reservation.
